# A Beautician's Dystonia: Long-Lasting Effect of Botulinum Toxin

**DOI:** 10.1155/2014/686181

**Published:** 2014-07-16

**Authors:** Siria Di Martino, Stefania Dalise, Giuseppe Lamola, Martina Venturi, Bruno Rossi, Carmelo Chisari

**Affiliations:** Unit of Neurorehabilitation, University Hospital of Pisa, 56124 Pisa, Italy

## Abstract

Treatment options for dystonia are not curative but symptomatic; the treatment of choice for focal dystonias is repeated botulinum toxin injections. Here, we present the case of a 46-year-old beautician with focal dystonia in her left hand that affected her ability to work. Pharmacological treatment with clonazepam and gabapentin failed to resolve her symptoms and was discontinued due to side effects (sleepiness, gastrointestinal disorders). Intramuscular injection of botulinum toxin (incobotulinumtoxinA, Xeomin) into the extensor digitorum communis (35 U), flexor carpi radialis (35 U), and flexor digitorum superficialis (30 U) muscles resulted in complete resolution of symptoms at clinical assessments at 1, 3, 6, and 10 months after the injections, confirmed by the results of surface electromyography 10 months after treatment. The patient was able to work again 1 month after treatment. No reinjection has been necessary at the last evaluation (12 months after treatment). In conclusion, botulinum toxin is an effective treatment for focal dystonia that can have long-lasting effects and can improve patients' ability to work and quality of life.

## 1. Introduction

Dystonia is the third most common movement disorder (annual incidence: 15–25 per 100,000), after Parkinson disease and tremor [1]. Herman Oppenheim first used the word “dystonia” in 1911 to describe a disorder causing variable muscle tone and recurrent muscle spasm [2]. In 1984, an ad hoc committee of the Dystonia Medical Research Foundation defined dystonia as a syndrome of involuntary, sustained muscle contractions affecting one or more sites of the body, frequently causing twisting and repetitive movements or abnormal postures [3]. There are several classifications of dystonia, based on genetic background, anatomical distribution, age at onset, and neurodegenerative processes [4]. The one most used in clinical practice is aetiological classification, which includes primary dystonia (idiopathic), secondary dystonia, dystonia-plus syndromes, and paroxysmal dystonia. Dystonia which affects only a single area is known as focal dystonia [5]. It can involve any part of the body, but it most often occurs in the neck (cervical dystonia), the eyelids (blepharospasm), or hands (task-specific or focal hand dystonia) [1]. Most focal dystonias are primary, though secondary forms are also known [6].

The onset of dystonic focal symptoms is usually insidious, beginning with a feeling of discomfort or tension in the limb when performing particular activities. Over time, usually within months to years, abnormal posture becomes apparent [7]. The severity and quality of dystonic postures may vary with body position, specific tasks, emotional state, or level of consciousness [8], but progression of symptoms is dependent on the specific patient.

The pathophysiology of focal dystonia features the excessive cocontraction of agonist and antagonist muscles. It can be detected by electromyography (EMG) of the involved muscles, which show abnormally prolonged activation bursts [9]. Of the different therapies used in focal dystonia, injections of botulinum toxin A (BTX-A), a neurotoxin that is extensively used to treat a variety of movement disorders, are the current treatment of choice [10]. BTX-A is injected into affected muscles with the aim of reducing the excessive contraction [9]. Its effect is maintained for approximately 8 weeks after which it gradually becomes inactive [11].

In this paper we report the case of a patient with a focal hand dystonia exacerbated by her work activity. Dystonic symptoms disappeared after an injection of 100 U incobotulinumtoxinA (botulinum neurotoxin free from complexing proteins; Xeomin; Merz Pharmaceuticals GmbH, Frankfurt, Germany) and the beneficial effect lasted for about 12 months after the treatment.

## 2. Case Presentation

A 46-year-old woman presented with discomfort in her left hand and difficulty in carrying out particular activities, especially during her job as a beautician. The problem evolved rapidly with appearance of abnormal posture of the left hand during repetitive movement performed at work, sometimes associated with pain. This condition severely limited her business. Previous examinations, including brain and cervical magnetic resonance scanning, had excluded a peripheral or central nervous disease as the cause of her symptoms. The patient's medical history excluded the presence of any psychiatric disorder. She has no story of psychogenic disturbances and she had never taken any antidepressants or neuroleptics. EMG of her forearm muscles showed the presence of dystonic activation patterns, indicating a diagnosis of focal dystonia. Pharmacological treatment with clonazepam and gabapentin was prescribed; however, as this failed to ameliorate the symptoms and caused side effects (such as drowsiness and gastrointestinal disorders), the patient suspended pharmacological treatment and came to our attention.

**Figure 1:**
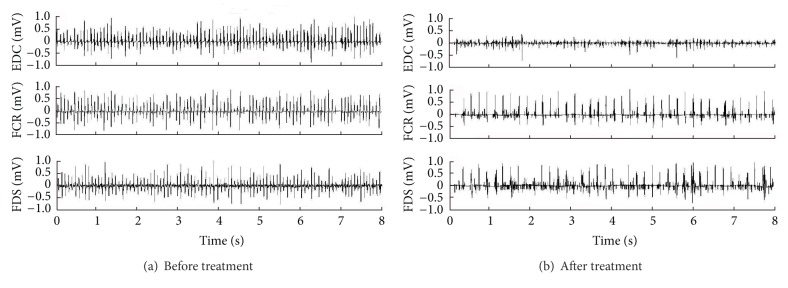
Surface EMG at rest (a) before treatment with botulinum toxin A and (b) 10 months after treatment. EDC: extensor digitorum communis; FCR: flexor carpi radialis; FDS: flexor digitorum superficialis.

When she came to our attention, the clinical examination showed an abnormal posture of the left hand with hyperextension of the IV and V finger and flexion of the interphalangeal joints of II and III finger. This particular posture was more evident during the execution of movements similar to those performed during her job. We performed surface EMG that confirmed the diagnosis of focal dystonia: her muscles showed typical signs of hyperactivity (Figure 1(a)). We administered 100 U incobotulinumtoxinA diluted in 1 mL saline solution into the following muscles of her left hand: extensor digitorum communis (35 U), flexor carpi radialis (35 U), and flexor digitorum superficialis (30 U). Electrical muscle stimulation was used to guide the injections.

One month later a marked reduction in muscle hyperactivity was observed. The patient mentioned a clear-cut reduction of discomfort and disappearance of the dystonic posture at as little as a few days after the injection and was able to work again. We conducted clinical assessments at 1, 3, 6, and 12 months after the injection and each time observed complete abolition of the symptoms. Moreover, after 10 months, we recorded surface EMG activity again, confirming the beneficial effect of incobotulinumtoxinA (Figure 1(b)). No reinjections have been necessary, up until the final clinical evaluation, performed 12 months after the original injection.

## 3. Discussion

This is an atypical form of primary dystonia. In early stages dystonia was exclusively linked to repetitive movement, while later the patient reported the symptoms even at rest.

To our knowledge this is the first case involving botulinum neurotoxin in the treatment of this kind of focal dystonia with such a long-lasting effect.

Generally, the benefit starts 1 week after the injection and lasts for roughly 3 months. The effect may be sustained for years with further injections [7, 10], so patients with dystonia usually need repeated botulinum toxin type A injections. Interestingly, Vecchio et al. [12] described dystonia in a musician who was treated with a botulinum toxin injection and received a beneficial effect lasting for 8 months. This patient needed a reinjection before being completely symptom-free.

The important findings which emerged in our case are the complete resolution of symptoms after a single injection and the very long-lasting effect. To optimise the effect of botulinum toxin type A injections it is necessary to achieve sufficient weakness to reduce spasm without hampering function. The lowest effective doses of botulinum toxin should be given in order to achieve these goals, and the injection site should be precisely identified [13]. We wish to underline that with this in mind we performed resting and dynamic surface EMG before treatment and used muscle stimulation to locate muscles accurately for injection.

## 4. Conclusions

In conclusion, we confirmed that incobotulinumtoxinA is an effective and well-tolerated agent for the treatment of focal dystonia. If it is used appropriately this approach can rapidly and for long periods relieve discomfort and provide a positive impact on patients' quality of life.
